# The scientific foundation for tobacco harm reduction, 2006-2011

**DOI:** 10.1186/1477-7517-8-19

**Published:** 2011-07-29

**Authors:** Brad Rodu

**Affiliations:** 1 Tobacco Harm Reduction Research, University of Louisville, Room 208, Clinical Translational Research Building, 505 S. Hancock Street, KY 40202, Louisville, USA

## Abstract

Over the past five years there has been exponential expansion of interest in tobacco harm reduction (THR), with a concomitant increase in the number of published studies. The purpose of this manuscript is to review and analyze influential contributions to the scientific and medical literature relating to THR, and to discuss issues that continue to stimulate debate. Numerous epidemiologic studies and subsequent meta-analyses confirm that smokeless tobacco (ST) use is associated with minimal risks for cancer and for myocardial infarction; a small increased risk for stroke cannot be excluded. Studies from Sweden document that ST use is not associated with benign gastrointestinal disorders and chronic inflammatory diseases. Although any form of nicotine should be avoided during pregnancy, the highest risks for the developing baby are associated with smoking. It is documented that ST use has been a key factor in the declining rates of smoking and of smoking-related diseases in Sweden and Norway. For other countries, the potential population health benefits of ST are far greater than the potential risks. In follow-up studies, dual users of cigarettes and ST are less likely than exclusive smokers to achieve complete tobacco abstinence, but they are also less likely to be smoking. The health risks from dual use are probably lower than those from exclusive smoking. E-cigarette users are not exposed to the many toxicants, carcinogens and abundant free radicals formed when tobacco is burned. Although laboratory studies have detected trace concentrations of some contaminants, it is a small problem amenable to improvements in quality control and manufacturing that are likely with FDA regulation as tobacco products. There is limited evidence from clinical trials that e-cigarettes deliver only small doses of nicotine compared with conventional cigarettes. However, e-cigarette use emulates successfully the cigarette handling rituals and cues of cigarette smoking, which produces suppression of craving and withdrawal that is not entirely attributable to nicotine delivery. THR has been described as having "the potential to lead to one of the greatest public health breakthroughs in human history by fundamentally changing the forecast of a billion cigarette-caused deaths this century."

## I. Introduction

In 2006 the American Council on Science and Health (ACSH) became the first American scientific organization to formally endorse tobacco harm reduction (THR), which involves the substitution of far safer sources of nicotine by those smokers who are unable or unwilling to achieve nicotine/tobacco abstinence. ACSH's position was based on a comprehensive review of the existing scientific and medical literature, which documented that (a) epidemiologic studies showed that smokeless tobacco (ST) use was at least 98% safer than smoking, (b) use of ST among men in Sweden was a major factor in very low prevalence of smoking, (c) ST use is not a gateway to smoking, (d) American smokers are misinformed about the scientific and medical basis for THR [1].

Among developed countries the U.S. currently provides the best access to a wide variety of smoke-free products, including traditional smokeless tobacco, Swedish snus, dissolvable tobacco (sticks, strips, orbs and pellets), e-cigarettes, and pharmaceutical nicotine (gum, lozenges, patches, nasal spray). Many other countries prohibit almost all of them, which sadly enhances the continued dominance of cigarettes. Development and marketing of alternative smoke-free nicotine delivery systems will be accelerated by recent acquisitions by major tobacco manufacturers. In 2009 Reynolds American Inc. purchased Niconovum, a Swedish company developing novel nicotine products. In 2011 British American Tobacco established Nicoventures Limited, which will develop and commercialize innovative nicotine products, and Philip Morris International purchased global patent rights to a nicotine-containing aerosol developed by scientists at Duke University.

Over the past five years there has been exponential expansion of interest in THR from medical and public health professionals, with a concomitant increase in the number of published studies. The purpose of this manuscript is to review and analyze influential contributions to the scientific and medical literature relating to THR, and to discuss issues that continue to stimulate debate.

The majority of studies published since 2006 have focused on ST use. Section II reviews both primary epidemiologic studies and meta-analyses that provide further evidence that the health risks from ST use are much lower than those from smoking and are extremely small in absolute terms. In contrast to these facts, Section III describes how misinformed Americans are about the relative health risks from smoking as compared with ST use. Section IV reviews new population-level evidence from Sweden, Norway and the United States suggesting that ST is an effective substitute for cigarettes, and also discusses evidence from clinical trials. Section V reviews studies from Sweden and the U.S. documenting that ST use is not a gateway to smoking, most notably among adolescents. Section VI discusses dual use of ST and cigarettes. Section VII reviews scientific studies of e-cigarettes--nicotine-delivery devices that have become popular despite bans in many jurisdictions. The growing global discussion of THR is discussed in Section VIII.

ST is used in many countries around the world, including India and others in South Asia [2]. Compared with manufacturing practices in Sweden and the U.S., there is little control over fermentation and curing in these regions, which may result in elevated levels of tobacco-specific nitrosamines (TSNAs) and other unwanted contaminants [2]. In addition, ST is often combined with betel leaf (Piper betle), sliced areca nut (Areca catechu) and/or powdered agricultural lime [2], which enhance the toxicity as well as the psychotropic effect of tobacco. These differences also result in higher morbidity and mortality among ST users in South Asia. This manuscript focuses exclusively on ST use in Western societies like Sweden and the U.S.

## II. Smokeless Tobacco Use Is Associated With Minimal Health Risks

### A. Cancer

In 2007, the International Agency for Research on Cancer published Monograph 89 [2], which contained the findings from an IARC working group established in 2004. The committee found that there was sufficient evidence that ST use is carcinogenic to humans, with sufficient evidence that "ST causes cancers of the oral cavity and pancreas."

It is important to understand the magnitude of the risks codified by Monograph 89. Unfortunately, although the document contained detailed descriptions of some epidemiologic studies relevant to cancer among ST users, the monograph did not present any summary risk estimates. Instead, some individuals from the working group, led by IARC epidemiologist Paolo Boffetta, published a meta-analysis in 2008 concluding that ST use was associated with elevated risks for cancers of the oral cavity (Relative risk, RR = 1.8), esophagus (RR = 1.6) and pancreas (1.6), all of which were statistically significant [3] (Table 1).

**Table 1:** Summary Relative Risks (95% Confidence Interval) for Smokeless Tobacco Use and Cancer From Two Meta-Analyses

Cancer (n = Boffetta: Lee-Hamling estimates)	Boffetta et al.	Lee-Hamling
		
Oral Cavity		
All (11:41)	1.8 (1.1 - 2.9)	1.79 (1.36 - 2.36)
Adjusted for smoking (0:19)	NA	1.36 (1.04 - 1.77)
Adjusted for smoking/alcohol (0:10)	NA	1.07 (0.84 - 1.37)
		
Esophagus		
All	1.6 (1.1 - 2.3)	1.25 (1.03 - 1.51)
Adjusted for smoking	NA	1.13 (0.95 - 1.36)
		
Pancreas		
All (6: 7)	1.6 (1.1 - 2.2)	1.00 (0.68 - 1.47)
Adjusted for smoking (0:7)	NA	1.07 (0.71 - 1.60)
		
Lung		
All (5: 9)	1.2 (0.7 - 1.9)	0.96 (0.73 - 1.27)
Adjusted for smoking (0: 6)	NA	0.99 (0.71 - 1.37)

NA, Not Available

Source: Boffetta et al. [3]; Lee and Hamling [4].

Even though these risks are low in comparison with smoking, a 2009 meta-analysis published by Peter Lee and Jan Hamling did not confirm them [4]. In fact, after appropriate adjustment for confounding factors, the Lee-Hamling analysis found that ST use was associated only with prostate cancer (Tables 1 and 2). Lee and Hamling commented that "Prostate cancer is not considered smoking related [original citations removed], and more information on its relationship with ST is needed before any clear conclusion can be drawn."

**Table 2:** Smoking-Adjusted Summary Relative Risks (RR) for Smokeless Tobacco Use and Other Cancers

Site (number of studies)	RR, (95% Confidence Interval)
	
Stomach (8)	1.03 (0.88 - 1.20)
Any Digestive (5)	0.86 (0.59 - 1.25)
Larynx (2)	1.34 (0.61 - 2.95)
Prostate (4)	1.29 (1.07 - 1.55)
Bladder (10)	0.95 (0.71 - 1.25)
Kidney (5)	1.09 (0.69 - 1.71)
Lymphoma (3)	1.35 (0.62 - 2.94)
	
All Cancer (7)	0.98 (0.84 - 1.15)

Source: Lee and Hamling [4].

Table 1 shows the major differences in risk estimates between the meta-analyses of Boffetta and that of Lee-Hamling for cancers of the oral cavity, esophagus and pancreas. A detailed subsequent analysis published by Lee and Hamling [5] revealed that the differences were due to the following factors: (a) Boffetta et al. did not use all available studies, and there were no specific criteria for which studies were included and which were excluded; Lee and Hamling used all available studies. (b) in some instances Boffetta et al. used only the highest risk estimates, even though they were derived from internally inconsistent subgroups of either ST exposure or of the disease outcome of interest; Lee and Hamling's analysis was conducted with clear and consistent criteria for ST exposure and disease outcomes [5].

The differences between the Boffetta et al. and Lee-Hamling meta-analyses are clearly illustrated in the case of pancreatic cancer, for which Boffetta reported a statistically significant summary RR of 1.6, and Lee-Hamling reported a summary RR of 1.07, which was not significant. The Lee-Hamling result was also reported in a more detailed analysis for pancreatic cancer published in 2008 by Sponsiello-Wang et al. [6].

Both meta-analyses used results from a 2005 Norwegian study [7] and a 2007 Swedish study [8] which reported risk estimates for pancreatic cancer among all snus users and for a subset of snus users who were never smokers. The Norwegian study reported a risk increase among all snus users (RR = 1.7, CI = 1.1 - 2.5) but not for the subset of snus users who were never smokers (RR = 0.9, CI = 0.2 - 3.1) [7]. The Swedish study reported exactly the opposite: There was virtually no risk among all snus users (RR = 0.9, CI = 0.7 - 1.2), but the subset of snus users who never smoked had an increased risk (RR = 2.0, CI = 1.2 - 3.3) [8].

As Lee pointed out in a recent review [9], "For pancreatic cancer, Boffetta cited only the increases for never smokers from the [Swedish] study and for the whole population from the [Norwegian] study, not mentioning the lack of increase for the whole population for the construction workers and for never smokers for the Norway cohorts." It is important to note that Boffetta was a co-author of both studies, which makes his selective use of data from them even more perplexing.

Ironically, the RR differences for pancreatic cancer between the Boffetta et al. and Lee-Hamling meta-analyses have been resolved in favor of Lee-Hamling by a third meta-analysis published in 2011, and it was co-authored by Boffetta [10]. It reported summary RRs for ever ST users (0.98, CI = 0.75 - 1.27), exclusive ST users (0.62, CI = 0.37 - 1.04), and ST users who smoked cigarettes (1.36, CI = 0.94 - 1.96). The authors concluded, "Our results on ST use are in broad agreement with a recently published meta-analysis of all published data on the issue, which reported no excess risk of pancreatic cancer in case-control studies" [6].

Other concerns have been raised about the validity of the risk estimates for pancreatic cancer from both the Norwegian [7] and Swedish [8] studies. The former was published in 2005 by Boffetta et al., and it was an extended follow-up of a cohort of Norwegian men recruited in the 1960s [7]. Boffetta et al. reported that ever users of ST had an increased risk for pancreatic cancer (RR = 1.67, CI = 1.12 - 2.50). However, despite the fact that alcohol consumption had been documented as the strongest risk factor for pancreatic cancer in this cohort, with odds ratios up to 10.8 [11], Boffetta et al. did not adjust the snus risk estimates for alcohol use [12]. In addition, Boffetta employed an unconventional classification for snus exposure and an unusual adjustment for smoking, as documented in a subsequent letter to the editor [12].

The Swedish study was published in 2007 by Luo et al., and it is one of many follow-up studies of Swedish construction workers published by investigators at the Karolinska Institute in Stockholm [8]. Luo et al. reported that current users of snus had an elevated risk for pancreatic cancer (RR = 2.1, CI = 1.2 - 3.6), but they excluded 135,000 construction workers recruited during the period 1971-74 because of "ambiguities" in questionnaire coding [13]. This cast considerable doubt about the credibility of a 1994 report by Bolinder et al., which stands almost alone in linking snus use with cardiovascular diseases [14]. The Bolinder cohort had contained only workers enrolled in the years excluded by Luo.

The Bolinder cohort [14] has been subjected repeatedly to inclusion and exclusion in ten studies published by Karolinska Institute investigators during the period 2005-2011 [8,15-23] (Table 3). The 2007 study by Hergens et al. [18], which found an elevated risk of fatal heart attack among snus users (RR = 1.28, CI = 1.06 - 1.55), is especially troublesome. This study excluded the Bolinder cohort, and Bolinder was a co-author, giving the impression that in 2007 Bolinder excluded her own cohort from 13 years earlier.

**Table 3:** Inclusion and Exclusion of the Bolinder Cohort in Karolinska Institute Studies

Year	First Author [Ref]	Bolinder In/Out	Major Findings (RR)
1994	Bolinder [14]	In	All CV disease (1.4), All causes (1.4)
2005	Odenbro [15]	In	Skin SCC (0.64)
2007	Luo [8]	Out	Pancreatic cancer (2.0)
2007	Odenbro [16]	In	Melanoma (0.65)
2007	Fernberg [17]	In	Leukemia, MM (0.81* - 1.24*)
2007	Hergens [18]	Out	MI (0.91*), fatal MI (1.28)
2008	Zendehdel [19]	In	Esophageal SCC (3.5) Non cardia stomach cancer (1.4)
2008	Hergens [20]	Out	Stroke (1.02*), fatal stroke (1.27*)
2008	Hergens [21]	Out	Hypertension (1.23 - 1.39)
2010	Carlens [22]	Out	Inflammatory diseases (0.9* - 1.1*)
2011	Nordenvall [23]	In	Colon, rectal, anal cancer (1.05*, 1.08*, 0.61*)

Ref = Reference number

RR = Relative Risk

CV = Cardiovascular

SCC = Squamous cell carcinoma

MM = Multiple myeloma

MI = Myocardial infarction

* Not Statistically Significant.

Hergens et al. [18] raised another serious and troubling question about Bolinder's characterization of snus use [14]. Bolinder defined "ST users" in her study as "subjects who reported only present ST use...," a clear representation of current use [14]. However, in describing the rationale for excluding the Bolinder cohort, Hergens et al. stated that "during the period 1971-1974 exposure information on snuff use was limited to ever or never use..." [18] The descriptions of snuff use in these two studies are conflicting and irreconcilable, and the only rational conclusion is that one is a misrepresentation. The credibility of the Karolinska Institute studies is contingent on the resolution of these discrepancies.

### B. Cardiovascular Diseases

There have been at least ten epidemiologic studies evaluating the risks for cardiovascular diseases (primarily heart attack and stroke) among ST users. Two meta-analyses by Lee in 2007 [24] and Boffetta and Straif in 2009 [25] have provided summary RRs from these studies.

#### 1. Heart attack

Both studies found that ST use is **not **associated with statistically significant elevated RRs for heart attack (RRs = 1.12, CI = 0.99 - 1.27; and 0.99, 95% CI = 0.89 - 1.10, respectively) [24,25]. However, Boffetta and Straif [25] reported an elevated risk for fatal cases among ever users (RR = 1.13, CI = 1.06 - 1.21), almost entirely derived from the Hergens study described previously [18] and a very large analysis in the U.S. conducted by the American Cancer Society on its first and second Cancer Prevention Studies [26].

Boffetta and Straif [25] did not find a dose-response effect for ST use and fatal heart attack, so the elevated risk from their study is somewhat tentative. In addition, although no elevated risks were observed in the majority of studies, the Cancer Society study [26] that reported elevated risks comprised 85% of the Boffetta-Straif analysis. This is noteworthy because smokeless users in this study also had elevated risks for emphysema (RR = 1.28, CI = 1.03-1.59) and lung cancer (RR = 2.0, CI = 1.23-3.24), two diseases closely associated with smoking. Thus, it is likely that there was residual confounding by smoking in the Cancer Society study that was responsible for heart attack risk among ST users.

#### 2. Stroke

The Lee [24] and Boffetta-Straif [25] meta-analyses also reported on the risk of stroke among ST users. Lee reported an increase in stroke risk among smokeless users (RR = 1.42, CI = 1.29 - 1.57)[24]. Boffetta and Straif [25] found no risk overall (RR = 1.19, CI = 0.97 - 1.47), but they found an elevated risk for fatal cases (RR = 1.40, CI = 1.28 - 1.54). Boffetta and Straif [25] did not find a dose-response effect for ST use and fatal stroke, so this risk is also somewhat tentative. The American Cancer Society study [26] comprised 89% of the Boffetta-Straif analysis, so the likelihood of smoking among smokeless users discussed in the previous paragraph is equally important for the elevated fatal-stroke risk.

In 2010, an analysis based on the Atherosclerosis Risk in Communities study reported that, compared with nonusers of tobacco, ST users had a slightly elevated incidence of cardiovascular disease events that was not statistically significant (Hazard Ratio, HR = 1.21, CI = 1.00 - 1.45) [27]. The HR was adjusted for confounders including age, sex, race, education, income, alcohol use, physical activity, smoking, blood pressure, diabetes, weight, and serum lipid levels.

In 2010 the American Heart Association released a policy statement on ST use. The statement was based on a literature review conducted by Piano et al. [28], which reported the following findings regarding various conditions:

Hypertension: "In summary, data from the majority of studies in this section do not support an increase in the incidence or prevalence of hypertension in ST product users."

Myocardial Infarction: "In summary, data derived from the majority of studies conducted in Sweden, whereby snuff/snus is the major ST product used, have not demonstrated a significant increase risk of nonfatal or fatal MI...Data derived from predominately U.S. populations are equivocal."

Stroke: "In summary, data from 2 studies (1 from the United States and 1 from Sweden) suggest that ST product use is associated with a slight increase in the risk of stroke mortality."

Other Cardiovascular Risk Factors: "Although the data are limited, most studies have found no relationship between ST use and other biochemical risk factors for [cardiovascular diseases]."

Thus, after a comprehensive review, the Piano et al. study showed that there were no markedly increased risks among ST users for cardiovascular disease. Nevertheless, the Heart Association policy position on ST was decidedly negative: "The American Heart Association does not recommend the use of ST as an alternative to cigarette smoking or as a smoking cessation product."

### C. Other Diseases

#### 1. Gastrointestinal Disorders

ST use is inevitably accompanied by swallowing of saliva that has mixed with tobacco extract, raising the possibility of an association with gastrointestinal (GI) disorders. A 2010 study was based on detailed GI symptoms obtained by a questionnaire distributed to 3,000 adults aged 18 to 80 years in the northern Swedish cities of Kalix and Haparanda relating to gastroesophageal reflux, dyspepsia (defined as pain above the stomach and/or nausea and feeling uncomfortably full after a meal), irritable bowel syndrome and other conditions [29].

About 1,000 survey respondents were subjected to endoscopic exams of their upper GI tracts, searching for ulcers of the esophagus, stomach and small intestine and for evidence of infection by *Helicobacter pylori*. Snus users (n = 96), smokers (n = 165) and dual users (n = 22) were compared with non-users (n = 432).

Snus users reported GI symptoms with the same frequency as non-users. In contrast, smokers were more likely to report dyspepsia (OR = 1.6, 95% CI = 1.1 - 2.2), and dual users were more likely to report dyspepsia (OR = 2.8, CI = 1.1 - 7.3) and IBS (OR = 3.3, CI = 1.3 - 8.2).

With respect to the endoscopy findings, snus users were no more likely than non-users to have ulcers of the esophagus, stomach or small intestine. Smokers were more likely to have peptic ulcer disease (OR = 2.3, CI = 1.0 - 5.2). The published paper presents detailed findings of numerous other minor studies that were performed. Compared with non-users, neither snus users nor smokers had elevated rates of Helicobacter infections.

In summary, the study offered reassurance that snus use is not associated with any significant GI symptoms or disorders.

#### 2. Parkinson's Disease and Multiple sclerosis (MS)

A 2005 study by the American Cancer Society showed that ST use may have been protective for Parkinson's Disease in the second Cancer Prevention Survey (RR = 0.22, CI = 0.07 - 0.67) [30].

There are no definitive causes of MS. In 2009 Hedström et al. published a population-based case-control study of tobacco use and MS [31]. It found that the OR for male smokers was 1.8 (CI = 1.3 - 2.5), and the OR for female smokers was 1.4 (CI = 1.2 - 1.7). The risk increased with the cumulative dose of smoking measured in pack-years (packs per day times years of smoking), which adds to the overall validity of the association. For example, compared to nonusers of tobacco, men who had up to 5 pack-years of smoking had an OR of 1.4 (CI = 1.0 - 2.0), while men who had 16 or more pack-years of smoking had an OR of 2.9 (CI = 1.7 - 5.1).

In contrast to smoking, the study found that snus users had lower risks of MS than nonusers of tobacco. These lower risks were present among snus users of 5 or more package-years who never smoked (OR = 0.4, not statistically significant) and who had smoked (OR = 0.3, CI = 0.1 - 0.9), the latter being statistically significant.

#### 3. Chronic Inflammatory Diseases

In 2010 Carlens et al. conducted a study of chronic inflammatory diseases among smokers and snus users in the Swedish construction workers cohort (Bolinder cohort excluded) [22]. The study reported that, compared with never users of tobacco, ever smokers had significantly elevated risks for rheumatoid arthritis (RR = 2.1, CI = 1.7 - 2.5), Crohn's disease (RR = 1.5, CI = 1.2 - 1.8), multiple sclerosis (RR = 1.9, CI = 1.4 - 2.6) and a lower risk for sarcoidosis (RR = 0.5, CI = 0.4 - 0.5), all of which echoed previous studies. Carlens et al. also reported that former smokers had an increased risk for ulcerative colitis (RR = 1.3, CI = 1.1 - 1.5), another recognized association. In contrast, ever use of snus was not associated with any of these diseases, although Carlens et al did not confirm the inverse association of snus use and MS seen in the report by Hedström et al. [31].

#### 4. Pregnancy Complications

One of the most common challenging questions regarding THR is whether it is applicable to pregnant women who smoke.

According to the 2004 Surgeon General's report, smoking during pregnancy is associated with increased risks for premature delivery, low-birth-weight infants, and stillbirth [32]. Smoking is also associated with increased risk for placental problems, including placenta previa and placental abruption, both of which can place the mother and fetus at risk. Paradoxically, pregnant women who smoke have a significantly lower risk for preeclampsia. But the overall effect of smoking on the developing fetus is decidedly negative.

Can a pregnant smoker who switches to ST benefit her health and that of her developing baby? Three studies have addressed this issue. The first, published in 2003 by England et al [33], reported information on pregnancy outcomes among Swedish women who used snus or smoked, compared with nonusers of tobacco.

In this study, tobacco users had smaller babies than nonusers, although the reductions were modest. The average baby weight for nonusers was 7 pounds 14 ounces; babies of snus users weighed 7 pounds 13 ounces, while light smokers (1-9 cigarettes per day) and heavier smokers (10 or more cigarettes per day) had babies that weighed less (7 pounds 8 ounces and 7 pounds 6 ounces, respectively).

Women who used snus were more likely than nonusers to have a premature delivery (adjusted odds ratio, aOR = 1.79, 95% confidence interval = 1.27 - 2.52), which was similar to that of light smokers (aOR = 1.56, CI = 1.33 - 1.83) and heavier smokers (aOR = 1.84, CI = 1.53 - 2.21).

This study also found that smoking is protective for preeclampsia. The aOR for light smokers was 0.71 (CI = 0.59 - 0.88), and heavier smokers' risk was even less (aOR = 0.48, CI = 0.36 - 0.64). However, snus users had a somewhat elevated risk for preeclampsia (aOR = 1.58, CI = 1.09 - 2.27).

In 2010 two studies from Wikström et al. also documented that snus use has risks for the developing fetus [34,35]. Both studies were based on over 600,000 pregnancies documented in the Swedish Medical Birth Register from 1999 to 2006.

The first study examined the effect of tobacco use on the risk for very premature (less than 32 weeks) or moderately premature (32-26 weeks) births [34]. It showed that snus users had a modestly elevated risk for a very premature birth (aOR = 1.38, CI = 1.04 - 1.83). The risk among light smokers (1-9 cigarettes per day) was 1.60 (CI = 1.42 - 1.81), and the risk among heavy smokers (10 or more cigarettes per day) was 1.90 (CI = 1.61 - 2.25). The study also showed that snus users had an elevated risk for a moderately premature birth of 1.25 (CI = 1.12 - 1.40), which was intermediate between light smokers (aOR = 1.18, CI = 1.12 - 1.24) and heavy smokers (aOR = 1.45, CI = 1.35 - 1.56).

The second study examined the effect of tobacco use on the risk for stillbirth [35]. It showed that women who were snus users had a modestly elevated risk (aOR = 1.57, CI = 1.03 - 2.41), which was again intermediate between light smokers (aOR = 1.15, CI = 0.91 - 1.45) and heavy smokers (aOR = 1.85, CI = 1.39 - 2.46).

This study did not confirm the elevated risk for preeclampsia seen in the 2003 report [33]. In addition, snus users did not have elevated risks for bleeding or for infants who were small for their gestational age, outcomes seen in both light and heavy smokers.

In summary, pregnant women who use snus are at risk for slightly smaller babies, and they also have modestly elevated risks for premature delivery, stillbirth and possibly preeclampsia. Although any form of nicotine should be avoided during pregnancy, the highest risks for the developing baby are associated with smoking.

### D. Summary of Health Effects

1. Primary epidemiologic studies and subsequent meta-analyses do not provide convincing evidence that ST use is associated with cancers of the oral cavity, pancreas, and gastrointestinal tract. There is evidence from one meta-analysis that ST use is associated with a small risk for prostate cancer, although a biologic mechanism for this disease has not been established.

2. Epidemiologic studies have not documented that ST use is associated with significantly elevated risks for myocardial infarction; however, a small increased risk for stroke cannot be excluded.

3. Studies from Sweden document that ST use is not associated with benign gastrointestinal disorders and chronic inflammatory diseases.

4. Pregnant women who use snus are at risk for slightly smaller babies, and they also have modestly elevated risks for premature delivery, stillbirth and possibly preeclampsia. Although any form of nicotine should be avoided during pregnancy, the highest risks for the developing baby are associated with smoking.

## III. Misperceptions of the Health Risks Associated with ST Use

It has been documented beyond question that, compared with smoking, ST use is associated with minimal health risks that are barely measurable by modern epidemiologic methods. However, this strong scientific rationale for THR is virtually unknown among the general public, and even among health professionals. Recent studies have shown the extent of these misperceptions and the potential impact they have on implementation of THR.

In 2007 Heavner et al. surveyed a convenience sample of 242 smokers in the Edmonton, Alberta area in conjunction with a test market of Swedish snus in the province [36]. About half of smokers had not considered switching to ST because they incorrectly believed that the health risks were the same as those associated with smoking, and about one-third thought that using ST would increase their risk for mouth cancer. A majority of smokers who had not considered switching because of ST risks, however, were willing to consider switching to a hypothetical reduced-risk product. Heavner et al. concluded that "many adult smokers are interested in switching to safer forms of nicotine, but the misperceptions [about nicotine and tobacco] are major barriers to harm reduction." In addition, they attributed the misperceptions "to the effective long-running disinformation campaign by anti-ST and anti-harm-reduction activists who are more concerned with promoting nicotine abstinence than public health."

In 2010 Peiper et al. published a study documenting widespread misperception of ST risks among highly educated university faculty at the University of Louisville [37]. They quantified the risk perceptions, among full-time faculty, of cigarette smoking and ST use with respect to general health, heart attack/stroke, all cancer, and oral cancer, comparing the results from faculty on the health science campus with those in schools not related to health.

Peiper et al. found that 51% of all faculty incorrectly believed that ST use confers general health risks that are equal to or greater than smoking. The misperception rate was lower for heart attack/stroke risk (33%) but higher for cancer (61%). The misperception rate for oral cancer was 86%. Although faculty on the health science campus had a somewhat lower rate than others (81% vs. 91%), the survey provided evidence that most health professionals have a poor understanding of the fact that ST use is vastly safer than smoking.

Peiper et al. believe that misperceptions result from "...anti-tobacco advocates and organizations..." that "conflate the risks of ST with the risks associated with cigarettes, using either direct or implied statements..."

Misperceptions about smoke-free products are present even in Sweden, where tobacco harm reduction has had a measurable impact on smoking. In 2010 Wikmans and Ramström reported that the majority of Swedish smokers have exaggerated perceptions of the harmfulness of pharmaceutical nicotine and snus, which they believe is an impediment to further reductions in smoking prevalence in that country [38].

In 2011 Callery et al. examined the perceptions and appeal of ST products, with and without pictorial health warning labels and relative health risk messages, among 611 Canadian smokers age 18-30 years [39]. They systematically varied the labels and messages on duMaurier snus, Marlboro snus, Copenhagen moist snuff and Ariva dissolvable tobacco products and measured the appeal of the products and participants' willingness to try them.

Callery et al. concluded: "The findings from the current study show relatively high levels of appeal for ST products and openness to trying ST products among young adult cigarette smokers in Canada... Pictorial warnings also exacerbated the false belief that smokeless products are equally as harmful as conventional cigarettes. Regardless whether ST products serve as a harm-reduction product at the population level, greater efforts should be undertaken to promote more accurate perceptions of relative health risks between tobacco products."

## IV. Evidence That ST is an Effective Substitute for Cigarettes

### A. Additional Evidence from Sweden

In 2006 Rodu and Godshall summarized the cumulative evidence from Sweden that ST, in the form of moist snuff called snus, has played an important role in low prevalence of smoking among Swedish men and, to a lesser extent, among Swedish women [1]. That year Ramström and Foulds reported the results from a population-based national Swedish survey from 2001-2002 [40]. They found that snus was the most common cessation aid among men, used by 24% during their last quit attempt. Among men who had used only one cessation aid, 58% had used snus, and the success rate among these subjects (66%) was significantly higher than that among those who used nicotine gum (OR = 2.2, CI = 1.3 - 3.7) or nicotine patch (OR = 4.2, CI = 2.1 - 8.6). Ramstrom and Foulds also found that "the odds of initiating daily smoking were significantly lower for men who had started using snus than for those who had not" (OR = 0.28, CI = 0.22 - 0.36). They concluded, "Use of snus in Sweden is associated with a reduced risk of becoming a daily smoker and an increased likelihood of stopping smoking."

In 2008 Furberg et al. investigated twelve variables and their interactions as correlates of smoking cessation among 14,700 regular smokers in the population-based Swedish Twin Registry [41]. They reported that ever use of snus was the strongest individual correlate for former versus current smoking (HR = 2.70, CI = 2.30 - 3.20). They concluded, "Swedes appear to be using snus as a form of nicotine replacement therapy despite a lack of clinical trials data to support its use as a smoking cessation aid."

#### 1. Population health effects

In 2007 Gartner et al. assessed the potential population health effects if snus was available in Australia (where it is now banned) [42]. They calculated the life expectancy among people with various trajectories of tobacco use, and they provided estimates of the net effects at the population level.

Gartner et al. summarized their findings: "There was little difference in health-adjusted life expectancy between smokers who quit all tobacco and smokers who switch to snus (difference of 0.1-0.3 years for men and 0.1-0.4 years for women). For net harm to occur, 14-25 ex-smokers would have to start using snus to offset the health gain from every smoker who switched to snus rather than continuing to smoke. Likewise, 14-25 people who have never smoked would need to start using snus to offset the health gain from every new tobacco user who used snus rather than smoking."

They concluded: "Current smokers who switch to using snus rather than continuing to smoke can realise substantial health gains. Snus could produce a net benefit to health at the population level if it is adopted in sufficient numbers by inveterate smokers."

In 2009 Rodu and Cole examined lung cancer mortality trends in European Union (EU) countries, starting from about 1950 and ending in 2002 [43]. Lung cancer is the sentinel disease of smoking, and a country's lung cancer mortality rate (LCMR) provides a reasonable indication of the amount of smoking in that country. The data came from the World Health Organization (WHO) and IARC.

In 2002, there were 172,000 lung cancer deaths among men in the EU. If all EU countries had the LCMR of men in Sweden, there would have been 92,000 fewer lung cancer deaths. Rodu and Cole also estimated that there were 509,000 smoking attributable deaths among men in EU countries in 2002. If all EU countries had the smoking rates of Swedish men, there would have been only 237,000 deaths, a reduction of 54% (Table 4).

**Table 4:** Deaths from Smoking in 2002 Among Men in EU Countries, and Deaths Based on Swedish Lung Cancer Mortality Rate

Country	All Deaths From Smoking in 2002	Deaths If Smoking At Swedish Rate	% Change At Swedish Rate
			
Austria	7,000	3,900	-44
Bulgaria	7,100	3,800	-46
Czech Republic	12,500	4,500	-64
Denmark	5,700	2,800	-52
Estonia	1,600	600	-66
Finland	4,100	2,600	-36
France	60,000	28,300	-53
Germany	83,700	43,700	-48
Greece	13,900	6,200	-56
Hungary	16,300	4,400	-73
Ireland	2,700	1,600	-43
Italy	75,300	34,200	-55
Latvia	2,600	900	-64
Lithuania	3,500	1,300	-63
Luxembourg	400	200	-53
Malta	400	200	-51
Netherlands	18,700	7,700	-59
Poland	48,500	14,400	-70
Portugal	7,000	5,100	-26
Romania	20,100	9,000	-56
Slovakia	4,900	1,900	-61
Slovenia	2,100	900	-58
Spain	46,100	21,100	-54
Sweden	5,200	5,200	---
UK	59,500	32,000	-46
All EU	509,000	236,500	-54

EU = European Union

UK = United Kingdom

Note: No data was available for Belgium and Cyprus.

Source: Adapted from Rodu and Cole [43].

The large differences in LCMRs between Sweden and other EU countries occur only in men. For most of the last 50 years, the LCMR among Swedish women was the sixth highest in the EU. This context is important, because it has been suggested that vigorous anti-smoking campaigns since the 1970s are the major determinant of the low Swedish smoking rates. However, it is implausible that these campaigns were highly effective for Swedish men and almost completely ineffective for Swedish women. The striking difference in the relative EU ranking of Swedish men and women is firm evidence that snus use, not anti-smoking campaigns, has played the primary role in low LCMR rates among men in Sweden for over a half century.

World War II created millions of male smokers, resulting in very high LCMRs throughout Europe in the 1960s and 1970s. Men in Portugal, Spain and Italy, which had LCMRs similar to those in Sweden in the early 1950s, later experienced peak LCMRs that were four to six times higher, while the peak in Sweden represented only a three-fold increase. Even though snus consumption declined until 1969, its use was high enough to suppress smoking by Swedish men and to keep their LCMR among the lowest in the EU. Increasing snus consumption in the last two decades has been accompanied by further declines in smoking. If current trends hold, the LCMR for Swedish men may become lower than that for Swedish women by 2011.

Currently, snus is banned in all EU countries except Sweden. While it cannot be proven that the availability of snus would reduce smoking prevalence in other EU countries, the study showed that snus use has had a profound effect on smoking among Swedish men for the past half century.

### B. Evidence from Norway

In 2008, the European Commission released a report entitled "Health Effects of Smokeless Tobacco Products." [44]. Except for one small part discussing THR (Section 3.8, pages 111-118), most of the report was very negative, even going so far as to deny that snus use has had any effect on smoking in Sweden and Norway.

The report concluded: "It is difficult to envision any significant impact of snus use on smoking cessation in Norway..." This was especially baffling, as Figures 19-22 (pages 42-43) show clearly that increased snus use over the last 20 years was concomitant with decreased smoking.

Norway occupies an interesting position in the European political arena, and in European tobacco issues. While it is located in the Scandinavian peninsula, shares a border with Sweden and has membership in the European Economic Area, Norway has twice rejected membership in the EU. Thus, it has not been subject to the EU ban on Swedish snus and similar smokeless products. In fact, information has emerged from Norway that the increasing use of snus in the past 20 years has resulted in a substantial decline in smoking among Norwegian men, a virtual reproduction of the Swedish experience reviewed by Rodu and Godshall [1].

Much of the information on THR in Norway has been produced by Dr. Karl Erik Lund, a respected tobacco researcher with SIRUS, the Norwegian Institute for Alcohol and Drug Research, an independent institution but also a government entity answerable to the Ministry of Health and Care Services.

In 2008, Dr. Lund gave a presentation on Norwegian tobacco use at the 51^st ^conference of the International Council on Alcohol and Addictions [personal communication]. He reported that among Norwegian men age 16-35 years, the prevalence of smoking declined from 50% in 1985, to 30% in 2007, while the prevalence of snus use increased from 10% to 30%.

Lund reported that snus is very popular as a quit-smoking aid among Norwegian men. Among those who quit smoking in 2007, snus was used by 23%, while nicotine gum was used by only 9%; the nicotine patch, Zyban and a quit line were used by even fewer (6%, 3% and 3% respectively).

Lund also presented information about the outcome of the last quit attempt by Norwegian male smokers age 20-50 years in 2007. Of those who used snus, 74% "quit smoking altogether" or experienced a "dramatic reduction in smoking intensity," which is very similar to the rate among American men in the 2000 National Health Interview Survey (NHIS) who switched to ST (discussed below in Section D). Lund added that the quit percentages for those who used nicotine gum, patch and Zyban were 50%, 47% and 40% respectively.

In 2009 Lund published a report on THR, in which he noted that existing anti-smoking measures will result in "diminishing marginal returns." [45] Lund provided a compelling rationale for THR, and noted in an epilogue that the Norwegian Health Directorate has cautiously endorsed the strategy.

In 2010 Lund et al. published a study confirming that Norwegian men prefer snus over all other methods to quit smoking [46]. The analysis was based on a survey by the Norwegian Institute for Alcohol and Drug Research, which asked 3,583 former or current smokers age 20-50 years what method they used when they last tried successfully (former) or unsuccessfully (current) to quit. Snus was used by 32% of all respondents, making it the most popular method by far. Other methods that enjoyed modest popularity were nicotine gum (18%), self-help material (12%), and the nicotine patch (10%). Nicotine inhaler, Zyban, Champix, telephone quit line, and help from health care professionals were also measured in the survey, but they had negligible usage rates.

Lund et al. reported an adjusted odds ratio (aOR) to indicate the effectiveness of ST products compared with nicotine gum, their reference product. For quitting completely, the aOR for snus was 2.7, meaning that it was nearly three times more effective than gum. Snus was also three times more effective than nicotine gum in "greatly reducing cigarette consumption" among continuing smokers (aOR = 3).

In 2011 Lund et al. also published a study comparing quit-smoking rates among snus users and never users in seven Norwegian surveys [47]. Quitting was defined as the percentage of ever smokers who were former smokers at the time of the survey.

As seen in Table 5, quit rates for snus users were always higher than for those who had never used snus; the results are statistically significant for all surveys except number 4. This provides compelling evidence that snus has played a powerful role in smoking cessation among Norwegians and, as Lund noted, it is consistent with the Swedish evidence.

**Table 5:** Quit Smoking Rates Among Snus Users and Never Snus Users in Seven Norwegian Surveys

Survey Number	Snus Users (%)	Never Snus Users (%)
		
1	80	52
2	55	23
3	81	63
4	62	53
5	75	45
6	90	50
7	73	43

Note: Compared with never snus users, snus users percentage statistically significant for all surveys except No. 4.

Source: Lund et al [47].

### C. Clinical Trials

Prior to 2006, only one clinical trial relating to THR had been conducted, a pilot study with one- and seven-year follow-ups [48,49]. But during the past five years, several more clinical trials have been completed.

In 2007 Mendoza-Baumgart et al. conducted small pilot trials focused on two ST products (either Exalt snus or Ariva dissolvable) versus a nicotine lozenge [50]. They evaluated toxicant exposure, subjective responses and product preferences among smokers using a cross-over design.

Mendoza-Baumgart et al. found that, compared with baseline smoking, all products produced significant reductions in 4-(methylnitrosamino)-1-(3-pyridyl)-1-butanol (NNAL), a metabolite of the TSNA 4-(methylnitrosamino)-1-(3-pyridyl)-1-butanone (NNK), and all produced comparable effects on withdrawal and craving. Ariva was the preferred product, followed by the nicotine lozenge and Exalt. The authors concluded that "These findings make it difficult to ignore the potential of some ST products, specifically Ariva, to reduce exposure to [TSNAs], particularly NNK..."

In 2008 Sharp et al. reported the results of a nurse-led smoking cessation program, employing ad libitum use of alternative nicotine products including snus, among 50 patients undergoing aggressive treatment for advanced stage head and neck cancer [51]. As they noted, "Our aim was to support the patients to be smoke-free during and after cancer treatment, rather than to be nicotine-free."

Sharp et al. reported that 74% of patients were confirmed cigarette abstinent during the treatment period. Forty-one patients were alive at the one-year follow-up, and 28 were off cigarettes. Multiple product use was common; the nicotine patch was the most commonly used product (91%), followed by snus (54%).

In 2008 Tønnesen et al. published the results of an open, randomized smoking cessation trial using group therapy and Oliver Twist, a ST pellet made in Denmark [52]. Cessation at 7 weeks was higher among subjects using ST (31.5% vs. 19.2%, OR = 1.94, CI = 1.05 - 3.62), but no significant differences were seen at the 6-month follow-up. At six months, 17.5% of participants were still using ST, even though a trial objective was cessation at 12 weeks after enrolment.

The unimpressive response rate for ST may have been influenced by negative attitudes among study staff. Tønnesen et al. commented that "...we do not believe that the therapists induced a positive expectation in the ST group. Our impression is that the nurses were not convinced that ST would help or would be accepted by the smokers." Nevertheless, they concluded that the trial demonstrated short-term efficacy of ST in combination with group therapy, and they indicated that other trials were underway.

In 2010 Caldwell et al. compared snus with nicotine gum among heavy smokers in New Zealand [53]. After observing smoking patterns and consumption for one (lead-in) week, Caldwell's group gave 63 smokers three different cigarette substitutes, each for two weeks. The substitutes were Swedish snus (4-gram pouches in three flavors), Habitrol nicotine gum (4 milligrams of nicotine) and a peppermint pouch containing 4 milligrams of nicotine embedded in microcrystalline beads, produced by a Swedish company called Niconovum.

The researchers collected information from the participants about the "acceptability and the willingness of smokers to use" the substitutes. They asked five questions gauging satisfaction, and they reported that "subjects scored Zonnic and snus more highly than gum for four out of the five..." All three products significantly reduced craving for cigarettes, and all three "...enabled subjects to reduce their smoking significantly compared with the lead-in week."

Participants ranked Zonnic and snus higher than nicotine gum for both quitting and reducing smoking. "At the conclusion of the study, subjects were asked to rank the three products in order of overall preference. For their first choice, an equal number (40%) chose snus or Zonnic, while 20% chose gum."

In 2010 Carpenter and Gray published a small but powerful study documenting that dissolvable tobacco products "led to a significant reduction (40%) in cigarettes per day, no significant increases in total tobacco use, and significant increases in two measures of readiness to quit, either in the next month or within the next 6 months." [54]

The authors randomly assigned 31 smokers who were uninterested in quitting to receive Ariva or Stonewall dissolvable smokeless products, or to continue smoking cigarettes. Smokers were given "minimal instructions on how to use" these products and were "told that there is no safe tobacco product and that the best thing they can do for their health is to quit entirely."

They wrote that their findings suggest "that Ariva and Stonewall are effective products to curb withdrawal and craving," and that there is "no evidence that ST (Ariva or Stonewall) undermines quitting. To the contrary, readiness to quit (in the next month and within the next 6 months) significantly increased among smokers who used a ST product relative to those who continued to smoke conventional cigarettes." This addressed the concern that telling smokers about vastly safer smokeless substitutes will "undermine quitting."

In 2010 Cobb et al. compared the acute effects (within 45 minutes) of administration of Ariva, Marlboro snus, Camel snus, and Commit lozenges with own-brand and Quest (very low nicotine) cigarettes to smokers who had been abstinent overnight [55]. Each of the 28 participants attended seven sessions, during which 40 physiological and subjective measures were assessed at several time points after two separate administrations of each product.

Camel snus and Commit significantly decreased an "intention to smoke" measure after the second administration, although not as much as own-cigarette brand. Ariva and Marlboro snus did not affect this measure. Very similar findings were also observed for a craving measure.

These results may have been related to the plasma nicotine levels produced by the various products. Own-brand cigarettes resulted in sharp and significant increases in plasma nicotine, and Camel snus produced a significant increase 15 minutes after the second administration, but the other products did not.

Although Ariva and Marlboro snus had no effect, Camel snus and Commit exhibited modest subjective effects on abstinence symptoms, even though they were not as effective as own-brand cigarettes. Nevertheless, Cobb et al. made the sweeping conclusion that "currently marketed non-combustible [potential reduced exposure products] may not be a viable harm reduction strategy for US smokers."

In 2011 Kotlyar et al. published the results of a clinical trial which was done in 2006-7, assessing whether RJ Reynolds' Camel Snus and Philip Morris' Taboka (a precursor of Marlboro Snus) were viable substitutes for cigarettes [56]. They recruited smokers who were interested in quitting, assigning them to use one of three products: 4 milligram nicotine gum or lozenge, Camel Snus or Taboka (participants had a choice of various flavors for each product). Participants were instructed to use at least one or two doses of the assigned product per day during a one-week sampling period; for the next four weeks, they were told to use the product at least 6 to 8 times daily (and additional doses if needed). During week 5, participants were required to reduce consumption of the substitute; by the end of that week they had to be completely tobacco- and nicotine-free.

There were several interesting results. First, all participants in all groups had a reduction in exhaled carbon monoxide, clearly demonstrating that they smoked less than before the study. Participants in all groups had a reduction in the urine concentration of N'-nitrosonornicotine (NNN) and NNAL. The reductions were statistically significant except for NNN in Camel Snus users (p = 0.07).

Overall craving and withdrawal scores decreased over the 4 weeks in all groups, with no differences between the groups. Continuous abstinence rates over the 4 treatment weeks varied from 33% (Taboka) to 43% (Camel Snus). Two weeks after the treatment ended, 39% of the Taboka group, 47% of the Camel Snus group and 56% of the nicotine group were not smoking, but these percentages dropped to 23%, 31% and 33% respectively after ten weeks of complete abstinence. One possible reason for the precipitous drop in the smoke-free percentages was the insistence on abstinence after 4 weeks.

There were other interesting aspects of this study. The Taboka group smoked significantly more than those using nicotine or Camel Snus. It is possible that Taboka, which had very low nicotine levels, simply didn't satisfy smokers.

A total of 1,159 smokers responded to advertisements for the study. According to the researchers, 800 "were able to be reached and were screened over the telephone," and 429 qualified and were interested in participating. Another 212 did not show up for the orientation. The attrition didn't stop there: 211 smokers were enrolled in the study but only 130 were randomized to one of the three groups. Just 80 participants completed the 4-week treatment period and the one-week transition to abstinence.

These numbers represent one of the biggest challenges of clinical trials, especially in the field of risky behaviors like smoking. The 80 participants who completed the study represent only 6.9% of the smokers who originally responded, so they are an especially motivated group. That has been one of the problems with quit-smoking trials: It is impossible to generalize their results because the subjects are almost always derived from a highly selected population that is not representative of smokers in general [57].

In 2011 Barrett et al. reported the effects of "Swedish-style" snus and a 2 mg nicotine lozenge on delay of smoking and craving reduction among 15 smokers using four double-blind placebo-controlled sessions [58]. The investigators, who conducted the study at Dalhousie University in Halifax, Nova Scotia, reported that snus delayed the urge to smoke and suppressed craving only in the eight men in the study, but that snus was ranked as the least preferred product.

Although the investigators acknowledged the impact on smoking and craving, they believed that "the therapeutic potential of [Swedish-style snus] may be limited by its acceptability." However, there are some elements of their protocol that may explain participants' dislike of the products. First, subjects "were told the products may contain some of the ingredients commonly found in cigarettes (e.g., tar, ammonia, carbon monoxide, menthol, nicotine, sucrose, etc.)." It is unclear how the investigators communicated that non-combustible products contained "tar" and "carbon monoxide," but these statement likely affected product ratings. Second, although the subject of the paper was Swedish snus, the investigators picked a brand made in Denmark instead of much more popular and successful Swedish products. Finally, the preference ratings were probably affected by the fact that the snus was unflavored, whereas the nicotine lozenge was flavored with mint.

In summary, the study by Barrett et al. showed that snus "may have some therapeutic potential for those attempting to quit smoking," but it was designed in a manner that portrayed a very negative view of its acceptability.

In 2011 O'Connor et al. reported the results of a 2008 sampling study of Camel snus, Marlboro snus, Stonewall dissolvable pellets and Commit nicotine lozenges among 59 smokers not interested in quitting, 44 of whom completed the entire study [59]. After all products were sampled, 45% of participants chose Commit, followed by Marlboro (29%), Camel (14%) and Stonewall (12%), with 80% very or somewhat likely to purchase their preferred product in the next year. Over the seven-day trial phase, cigarette consumption decreased from 11.8 to 8.7 per day while use of the other products was constant at 4.7 units per day. This was accompanied by a decline in exhaled carbon monoxide.

Despite these findings, O'Connor et al. made some very negative conclusions: "However, we observed no true switching (i.e., abandoning cigarettes), even though [smokeless] and [nicotine replacement] products were provided without cost. It is clear that simply informing smokers of the lower risk and providing products is not going to result in major immediate shifts to smokeless alternatives. In the absence of some significant incentive, it is unlikely that information campaigns alone would lead to migration from use of cigarettes toward less hazardous nicotine sources among United States smokers."

It is unclear how O'Connor et al. generalized the results from 44 subjects in Buffalo, NY to all American smokers. Furthermore, although two sessions "presented information about the relative risks of ST and nicotine replacement products compared to cigarettes to provide a health rationale for considering these products as alternatives," the paper did not disclose the details about the extent and tone of the information, which may have played an important role in the results. Twenty-three of the subjects were women, who may have had a pre-existing bias against ST. In addition, 23 had already used pharmaceutical nicotine, which may partially explain the preference for Commit.

O'Connor et al. provided a valuable insight: "...the greater the range of products offered, the greater the proportion of smokers who may find a product they see as a viable substitute for cigarettes. This is consistent with the body of literature [original references omitted] suggesting that smokers' varied reactions to different products may be informative in themselves, meaning a 'sampling' approach may allow smokers to find an appealing alternative product to cigarettes."

### D. American Survey Evidence

In 2008 Rodu and Phillips provided the first population-level evidence that American men have quit smoking by switching to ST [60]. Using data from the 2000 NHIS, which the CDC uses to estimate smoking prevalence in the U.S., Rodu and Phillips estimated that 359,000 American male smokers had tried to switch to ST during their most recent quit attempt, and 73% (261,000, termed switchers) were former smokers at the time of the survey, representing the highest proportion of successes among all methods. In comparison, the nicotine patch was used by an estimated 2.9 million men in their most recent quit attempt, but only 35% were former smokers at the time of the survey. Of the 964,000 men who had used nicotine gum, 34% became former smokers. Of the 98,000 men who used the nicotine inhaler, 28% quit successfully. None of the estimated 14,000 men who had tried the nicotine nasal spray became former smokers.

Rodu and Phillips showed that switching to ST compares very favorably with pharmaceutical nicotine as a quit-smoking aid among American men, despite the fact that few smokers know that the switch provides almost all of the health benefits of complete tobacco abstinence. The results of this study show that THR is a viable cessation option for American smokers.

In 2009 Biener and Bogen reported the results from the 2006-2007 Indiana Adult Tobacco Survey that had implications for THR [61]. Indianapolis had served as a test market of Swedish-style snus by the two largest American cigarette manufacturers. In 2006 Philip Morris launched a test market there for Taboka snus (in 2008 it was discontinued when Marlboro snus was launched). In 2007 Indianapolis was one of several expansion markets for RJ Reynolds' Camel Snus; that product went on to national distribution in 2009.

Biener and Bogen reported that almost 20% of survey respondents throughout Indiana were aware of snus. Awareness among smokers statewide was 44%, which was 4.5 times higher than awareness among non-smokers.

Awareness among respondents in central Indiana (i.e. around Indianapolis) was 29%. More importantly, about 64% of male smokers in central Indiana had heard about snus, and 20% had tried it. This is evidence that Philip Morris and Reynolds were targeting adult male smokers in their test-market campaigns, and that the manufacturers were fairly successful.

Biener and Bogen also reported that risk perception played an important role in getting people to try snus. Respondents who correctly believed that ST is less harmful than cigarettes were almost four times as likely to try snus as those who believed the misinformation about the differential risks. Unfortunately, this study revealed that 88% of all respondents incorrectly believed that ST was just as dangerous as cigarettes.

Biener and Bogen offer some perceptive comments on the sad state of smoker misinformation:

"Both marketing and health education messages should include the information that all tobacco products are harmful and that abstinence from all tobacco products is the most healthful choice. At the same time, simply saying that ST is 'not safe' is not a sufficient stance for public health communications. There is a recognized continuum of risk along which various tobacco products can be placed, with low-nitrosamine ST products much lower on the risk continuum than combustible tobacco, although it is not harmless. Devising an effective way to inform the public about the continuum should be an important research priority, as currently consumers are woefully incorrect in their assessments of relative risk of various tobacco products. This state of affairs could result in people deciding not to give up smoking in favor of a product lower on the risk continuum because they assume that all tobacco products are equally harmful."

### E. Summary

There is extensive research evidence that ST use has been a key factor in the declining rates of smoking and of smoking-related diseases in Sweden. While it cannot be proven that the availability of ST would reduce smoking prevalence in other countries, the potential population health benefits of ST are far greater than the potential risks. This makes the continued prohibition of ST in cigarette-dominant markets an unsustainable and counterproductive public health debacle.

## V. ST Use is Not a Gateway to Smoking

### A. Evidence from Sweden

In 2006 Rodu and Godshall reviewed the published studies from Sweden documenting that there is no evidence that ST is a gateway to smoking, especially among youth [1]. This was confirmed in a 2008 study of 3,000 adolescents from the Stockholm area by Galanti et al. [62]. They found that "the majority of tobacco users (70%) started by smoking cigarettes" and "the proportion of adolescent smoking prevalence attributable to a potential induction effect of snus is likely small."

In 2008, the European Commission's Scientific Committee on Emerging and Newly Identified Health Risks concluded that "The Swedish data...do not support the hypothesis that...snus is a gateway to future smoking." [44]

### B. Evidence from the U.S

Opponents of THR in the U.S. believe that it will lead to increased teenage ST use, which will function as a "gateway" to smoking [63]. It has been observed that teenagers who use ST are more likely than non-users to subsequently smoke [64-68]. But a close examination of the evidence suggests only that ST use is one of several behaviors associated with smoking, not that it leads to smoking.

In the U.S., concomitant use of cigarettes is common among ST users [69] (dual use will be covered in Section VI). However, investigators have not found credible evidence that ST use is a gateway to smoking among American youth. In 2003 Kozlowski et al. analyzed data from the 1987 NHIS survey and concluded that there was little evidence that ST use was a gateway to smoking, because the majority of ST users had never smoked or had smoked cigarettes prior to using ST [70].

The belief that ST is a gateway to smoking is based mainly on two longitudinal studies comparing subsequent smoking among adolescent ST users and non-users [68,71]. The first study, which used the 1989 Teenage Attitudes and Practices Survey and its 1993 follow-up, found that young males who used ST were significantly more likely to have become smokers at follow-up than non-users of tobacco (OR = 3.5, CI = 1.8 - 6.5) [68]. However, a subsequent analysis revealed that the earlier study did not take into account well-known psychosocial predictors of smoking initiation that were in the TAPS, including experimenting with smoking, below average school performance, household member smoking, depressive symptoms, fighting and motorcycle riding [72]. Inclusion of these variables into a multivariate model reduced the odds ratio of smoking among regular ST users to 1.7, which was not statistically significant. The investigators concluded that the earlier "analysis should not be used as reliable evidence that ST may be a starter product for cigarettes."

The second study found that 7^th ^and 9^th ^grade students who had used ST (in the past 30 days) were more likely than nonusers to be smoking two years later (OR = 2.6, 95% CI = 1.5 - 4.5), after controlling for smoking by family and friends, low grades, alcohol use and deviant behavior [71]. However, Timberlake et al. [73] have observed that regression analysis may not adequately control for imbalances in covariate distributions between ST users and nonusers. They analyzed data from the National Longitudinal Study of Adolescent Health after propensity score matching and found that adolescent ST use was not associated with an increased risk of smoking in later adolescence or young adulthood [73].

In 2005 O'Connor et al. examined data from the 2000 National Household Survey on Drug Abuse to determine if ST use led to smoking. They described the impact of ST use on subsequent cigarette smoking initiation as "minimal at best," and they concluded that the association of ST use and smoking seen in other reports "is likely a manifestation of dual experimentation rather than a causal relationship." [74]

In 2010 Rodu and Cole investigated the gateway issue by analyzing data from the 2003-05-07 National Survey on Drug Use and Health, which asked survey participants at what age they used cigarettes or smokeless for the first time [75]. Using this information, Rodu and Cole classified participants as cigarette initiators, ST initiators, or both, and they determined the prevalence of current smoking among these groups at the time of the survey. The analyses were restricted to white men age 18 years or over, and white boys aged 16 to 17 years, which are the groups most likely to have used ST.

The prevalence of current smoking among white men who were cigarette initiators was 35%. In comparison, the prevalence of smoking among ST initiators was only 28%, which was significantly lower (Prevalence ratio, PR = 0.80, CI = 0.77 - 0.84). The results for boys were even clearer. Current smoking among cigarette initiators was 43%, but only 18% of ST initiators smoked. This means that boys who had started with ST were less than half as likely to be smoking at the time of the survey (PR = 0.43, CI = 0.36 - 0.52).

Rodu and Cole concluded that "ST use has played virtually no role in smoking initiation among white men and boys, the demographic groups among which ST use is most prevalent. There is evidence that, compared with cigarette initiators, ST initiators are significantly less likely to smoke. This suggests that ST may play a protective role."

Despite the preponderance of the evidence, claims of a gateway effect persist, which prompted O'Connor et al. to note in 2005, "Continued evasion of the [harm reduction] issue based on claims that ST can cause smoking seems, to us, to be an unethical violation of the human right to honest, health-relevant information." [74]

### C. Summary

It is now established that ST use is not a gateway to smoking in Sweden, nor in the U.S. In fact, there is evidence that the opposite is true: ST users may play a protective role against subsequent cigarette smoking.

## VI. Dual Use of ST and Cigarettes

Dual use is the object of persistent complaints by opponents of THR. For example, in 2002 Henningfield et al. described the theoretical adverse consequences of dual use [76]. Despite their concerns, they acknowledged that "There are virtually no data that currently exist on the safety of such use or the degree to which such use will foster the perpetuation of smoking or contribute to reduced overall smoking...The issue warrants further study."

### A. Evidence

In 2010 that study was completed by Frost-Pineda et al, who reviewed 17 published research studies that had data on the health risks from dual use versus those from smoking [77]. Frost-Pineda and colleagues conclude that "...there are not any unique health risks associated with dual use of ST products and cigarettes, which are not anticipated or observed from cigarette smoking alone." The authors further commented that "some data indicate that the risks of dual use are lower than those of exclusive smoking."

Frost-Pineda et al. also reviewed longitudinal studies in the U.S. and Sweden to determine if dual users had a different trajectory of tobacco use and cessation than that of exclusive smokers. A 2002 study by Wetter et al. found that 11% of dual users were tobacco-abstinent after 4 years of follow-up, compared with 16% of exclusive smokers [69]. However, 80% of exclusive smokers were still smoking at the 4-year follow-up, while only 27% of dual users were smoking; 44% were still dual users and 17% were exclusive smokeless users.

Very similar results have been reported in longitudinal studies of dual users in Sweden. For example, Rodu et al. reported the follow-up tobacco status of men in northern Sweden who were either cigarette smokers or dual users when they enrolled in a population-based epidemiological study [78]. Among exclusive smokers followed for 5 years, 69% were still smoking, 4% were dual users, 7% used ST, and 19% were tobacco free; the respective percentages among dual users were 6%, 52%, 24%, and 18% [79]. Among smokers followed for 9 years, 51% were still smoking, 10% were dual users, 16% used ST, and 45% were tobacco free; the respective percentages among dual users were 4%, 44%, 41% and 11%. Among smokers followed for 13 years, 46% were still smoking, 7% were dual users, 12% used ST, and 36% were tobacco free; the respective percentages among dual users were 9%, 22%, 60%, and 9%.

Frost et al. concluded that, although dual users are less likely than exclusive smokers to be completely tobacco abstinent at follow-up, they are much less likely to be smoking.

### B. Summary

In follow-up studies, dual users are less likely than exclusive smokers to achieve complete tobacco abstinence, but they are also less likely to be smoking. Follow-up studies also suggest that the health risks from dual use are lower than those from exclusive smoking.

## VII. Electronic Cigarettes (E-cigarettes)

### A. Introduction

E-cigarettes are battery-powered devices that vaporize a mixture of water, propylene glycol, nicotine and flavorings. They are activated when the user inhales through the mouthpiece of the device. To date, most e-cigarettes and mixtures are manufactured in China.

E-cigarettes have been sold on the American market for several years. In 2008 and 2009, the U.S. Food and Drug Administration (FDA) detained shipments being imported by two American distributors, Smoking Everywhere and NJOY, on the grounds that the items were unapproved drug-delivery devices. The distributors filed a lawsuit in federal district court, and in January 2010 Judge Richard J. Leon ruled that the FDA does not have the authority to regulate e-cigarettes in that manner [80]. Judge Leon ordered that "the FDA shall not detain or refuse admission into the United States of [Smoking Everywhere's and NJOYs] electronic cigarette products on the ground that those products are unapproved drugs, devices, or drug-device combinations."

The judge found that the 2009 "Tobacco Act applies to 'tobacco products,' which Congress defined expansively as 'any product made or derived from tobacco that is intended for human consumption'...Congress enacted the Tobacco Act to confer FDA jurisdiction over any tobacco product - whether traditional or not - that is sold for customary recreational use, as opposed to therapeutic use. As such, the Tobacco Act, in effect, serves as an implicit acknowledgment by Congress that FDA's jurisdiction over drugs and devices does not, and never did, extend to tobacco products, like electronic cigarettes, that are marketed in customary fashion for purely recreational purposes."

Finding that e-cigarettes, like all tobacco products, are subject to FDA oversight but fall outside of both drug and device categorization, Judge Leon characterized the FDA's attempt to apply pharmaceutical standards to e-cigarettes as "bootstrapping run amuck."

The FDA filed an appeal, and in January 2011 an appellate court affirmed Judge Leon's decision requiring the FDA to regulate e-cigarettes as tobacco products, rather than as drug-delivery devices. On April 25, 2011 the FDA accepted the appellate ruling. In an open letter published on the agency's website, Tobacco Center Director Lawrence R. Deyton and Drug Center Director Janet Woodcock acknowledged that e-cigarettes are tobacco products and would be subject to regulations under the 2009 Tobacco Act [81].

The FDA decision was a victory on several counts for American smokers and for public health. First, the FDA decision guarantees that e-cigarettes, which have helped many smokers quit, will remain on the market. Second, the Deyton-Woodcock letter indicated that FDA regulation of e-cigarettes will subject them "to general controls, such as registration, product listing, ingredient listing, good manufacturing practice requirements, user fees for certain products, and the adulteration and misbranding provisions, as well as to the premarket review requirements for 'new tobacco products' and 'modified risk tobacco products.'" These requirements will promote the marketing of safe and quality-controlled products. Finally, the decision could allow pharmaceutical companies to reposition nicotine medicines as recreational alternatives to cigarettes. Today, these products are sold with a therapeutic claim for smoking cessation, but they are expensive, unsatisfying and FDA-approved only for temporary use (10-12 weeks). That accounts for their dismal success rate among smokers. Pharmaceutical companies may enter the recreational nicotine market with products that satisfy smokers indefinitely and are cheap enough to compete directly with cigarettes. Clearly, the tobacco industry is poised to compete in this market -- Reynolds American owns Niconovum (here http://www.niconovum.se/ ) and British American Tobacco recently formed Nicoventures (here http://www.nicoventures.co.uk/).

### B. Scientific Studies of E-cigarettes

#### 1. Clinical Studies

Cigarette smoke contains thousands of chemical agents in addition to nicotine. In comparison, e-cigarettes produce a vapor consisting primarily of water, propylene glycol, nicotine and flavorings. The ingredients themselves do not pose any significant health risks. Nicotine is one of the most intensively studied drugs in history; while it is highly addictive, it is not the primary cause of any of the diseases related to smoking. Propylene glycol is approved by the FDA for use in a large number of consumer products. It is sometimes vaporized, forming artificial smoke in theatrical and other productions. While brief exposure to propylene glycol vapor is not associated with any adverse health effects, there are no studies relating to long-term daily exposure.

Nevertheless, tobacco control activists have aggressively attacked these products. For example, in 2009 Dr. Jack Henningfield, a scientific adviser on tobacco to the WHO and an advisor to GlaxoSmithKline on pharmaceutical nicotine, called e-cigarettes "renegade products" for which "we have no scientific information." [82] He then stated that e-cigarettes "are not benign," although there was no explanation in his article of how he came to that conclusion in the absence of any scientific information. While it is true that there is a paucity of scientific studies pertaining to this new area, and that the discussion has become highly polarized, several reports have provided important information.

There has been almost no research conducted on the absorption of nicotine from e-cigarettes and its distribution across various anatomic sites, but a study of the pharmaceutical nicotine vapor inhaler is informative. In 1995 Bergström et al. asked smokers to use an inhaler containing radiolabeled nicotine by using shallow frequent "puffing" inhalations or deep "pulmonary" inhalations, and they measured nicotine uptake by positron emission tomography (PET) [83]. They found that participants had about 45% of the inhaled nicotine in the oral cavity and pharynx, 10% in the esophagus (suggesting transfer from the oral cavity) and 8% in the respiratory tract. The rest of the nicotine was probably in sites that were not covered by the PET scan. No significant differences were noted between the types of inhalation. The results of this trial have important implications for e-cigarette users: they are probably absorbing nicotine mainly from the oral and pharyngeal mucosa.

In 1995 another study by Lunell et al. found that 80% of participants preferred shallower puffing for nicotine inhalers, and only 13% favored deeper inhalation [84]. They also observed that a placebo inhaler containing no nicotine resulted in partial suppression of some withdrawal symptoms such as irritability and difficulty in concentrating.

In 2010 Bullen et al. published the results of a crossover clinical trial in which e-cigarettes containing 0 mg. and 16 mg. of nicotine were compared with a nicotine inhaler and own-brand cigarettes among 40 smokers who had been abstinent overnight [85]. Bullen et al. reported that 16 mg. e-cigarettes and the inhaler produced the same reduction in desire to smoke and other withdrawal symptoms. Both products produced modest elevations in peak blood nicotine (1.3 and 2.1 ng/ml respectively) that were much lower than that produced by cigarettes (13.4 ng/ml). Compared with inhalers, e-cigarettes resulted in significantly less frequent mouth and throat irritation (88% vs. 38%). Bullen et al. concluded that the tested e-cigarettes were "well tolerated, acceptable to most users, rated significantly more pleasant to use than the inhalator, and in the first hour exhibited a pharmacokinetic profile more like the inhalator than a tobacco cigarette, without excess adverse events. These findings suggest potential to help people stop smoking in the same way as a nicotine inhalator."

In 2010 Eissenberg used a controlled puffing regimen and compared two brands of e-cigarettes with own-brand cigarettes, measuring blood nicotine levels, heart rate and craving among 16 smokers abstinent for 12 hours [86]. He concluded that the e-cigarettes "delivered little to no nicotine," and the measured increases in blood nicotine were very similar to those from Bullen et al. In addition, Eissenberg found that e-cigarettes "suppressed craving less effectively," although both brands produced reductions, one of which was significant at a single time point. Later in 2010 the same research group included these results in an expanded trial that included 32 smokers, but the conclusions remained essentially the same [87].

In 2010 Trtchounian et al. used a smoking machine to compare the puffing characteristics and smoke/aerosol densities of 4 brands of e-cigarettes, Liberty Stix, Smoking Everywhere, NJOY and Crown Seven with those of combustible cigarettes [88]. They found that, except for Liberty Stix, e-cigarettes required a much higher vacuum for puffing than commercial cigarettes; the vacuum required for the former brand was the lowest of all tested products. In addition, puff strength had to be increased as puff number increased. They also found considerable variation in the maximum number of puffs delivered by the products, which ranged from 177 (Smoking Everywhere) to 313 (NJOY).

Recent research documents that it is possible to deliver satisfying doses of nicotine via inhalation. In 2010 Rose et al. described a pilot study of vaporized nicotine pyruvate among nine overnight-abstinent smokers [89]. Rose et al. determined that nicotine pyruvate delivers more effective pulmonary doses than pure nicotine because vapor particle size is smaller (0.6 *u*m) than other nicotine inhalers, and the neutral pH of the solution is less irritating. Compared with placebo, nicotine pyruvate inhalation produced a sharp increase in plasma nicotine levels and was moderately satisfying to smokers. Rose et al. concluded that nicotine pyruvate "has promise as a potentially more effective form of nicotine replacement. The pharmacokinetic and subjective data demonstrated that this technology can be used to administer nicotine by the pulmonary route for rapid absorption, coupled with acceptable sensory qualities, to provide subjective satisfaction and relief of craving." They also commented about "long term nicotine replacement, to be used by smokers who would otherwise relapse to smoking; this approach would be analogous to methadone maintenance, which has been demonstrated to be an effective treatment of heroin addiction (reference omitted). In this harm reduction scenario, ex-smokers would continue to receive the perceived benefits of nicotine while minimizing the risk of disease from combustion and pyrolysis products, including nitrosamines, polycyclic aromatic hydrocarbons, carbon monoxide and numerous other toxic substances contained in tobacco smoke."

#### 2. Laboratory Studies

In 2008 Health New Zealand released the results of a comprehensive battery of laboratory tests on Ruyan e-cigarette liquid [90]. This organization found that cartridges contained TSNAs at trace levels, approximately 4 parts per billion. It evaluated mist samples for well characterized toxic agents in cigarette smoke. The samples did not contain detectable levels of 1,3-butadiene, acrolein, acrylonitrile, benzene, ethylene glycol, ethylene oxide and hydrogen cyanide. The following trace levels of other agents included (parts per million): acetaldehyde (0.34), acetone (0.16), formaldehyde (0.25), cresol (0.16), xylene (0.18) and styrene (0.29). Health New Zealand also found traces of some of the 34 polycyclic aromatic hydrocarbons that it surveyed, but it found no detectable heavy metals.

On July 22, 2009, the FDA released the results of laboratory tests of e-cigarettes, which were conducted by the Division of Pharmaceutical Analysis at the FDA's Center for Drug Evaluation and Research [91]. In a press release, the FDA said: "These tests indicate that these products contained detectable levels of known carcinogens..."

The FDA analyzed 18 cartridges from two e-cigarette manufacturers, Smoking Everywhere and NJOY [91]. The FDA analyzed 14 products from Smoking Everywhere, but the agency only reported the TSNA levels for 7 of those products; it tested 3 out of 4 NJOY products. It is not clear why the FDA tested only half of the company's products for carcinogens, nor how did the agency chose the products. There are some clues in the report. First, the products that weren't tested simply had blank boxes in the results chart. A footnote says, "Open boxes indicate the sample was not available for testing." Another note in the methods section admitted that "...not all sample lots were available for analysis...as they were consumed in other testing." The FDA didn't purchase enough of the products to conduct the testing in a systematic and scientific manner.

What the FDA didn't test is even more important than what the agency tested. The report noted that the "Nicotrol Inhaler, 10 mg cartridge was used as a control for some test methods." That inhaler is a pharmaceutical nicotine product that is regulated by the FDA, but the agency didn't test the product for TSNAs. This is a critical omission, because it's been known for over 20 years that nicotine medications contain TSNAs [92].

Why did the FDA analyze e-cigarettes for carcinogens, when there is no evidence the agency ever conducted carcinogen studies of products that they have regulated for over 20 years? Is it possible that the FDA approved medicines that contained TSNAs, but the agency is now disapproving e-cigarettes because they contain the same contaminants? To answer these important questions, we have to know how high - or how low - the TSNA levels are in these products.

Unfortunately, the agency did not report TSNA levels. Instead, it reported that TSNAs were either "Detected" or "Not Detected," which is entirely inadequate. Many tobacco products have TSNA levels in the single-digit parts per million range, a level at which there is no scientific evidence that they are harmful [93]. According to the report, the FDA used an analytic method published in 2008 [94]. The report notes that "the published method is quite sensitive for the TSNAs..." and it goes on to explain that the level of detection is 40 parts per trillion. Thus, it seems that the FDA tested e-cigarette samples using a method that detects TSNAs at about 1 million times lower concentrations than are conceivably related to human health.

In summary, the FDA tested e-cigarettes for TSNAs using a questionable sampling regimen and using methods that were so sensitive that the results are highly unlikely to have any possible significance to users. The agency failed to report specific levels of these contaminants, and it has failed to conduct similar testing of nicotine medicines that have been sold in the U.S. for over 20 years.

### C. Summary

E-cigarettes produce a vapor composed of water, propylene glycol and nicotine, so e-cigarette users are not exposed to the thousands of toxic agents formed when tobacco is burned. Although laboratory studies have detected trace concentrations of some contaminants, this appears to be a small problem that is amenable to improvements in quality control and manufacturing that are likely with FDA regulation as tobacco products. There is limited evidence from clinical trials that e-cigarettes deliver only small doses of nicotine compared with conventional cigarettes. However, e-cigarette use emulates successfully the cigarette handling rituals and cues of cigarette smoking, which produces suppression of craving and withdrawal that is not entirely attributable to nicotine delivery.

## VIII. The Growing Global Discussion of Tobacco Harm Reduction

In 2006, ACSH concluded "that strong support of THR is fully consistent with its mission to promote sound science in regulation and in public policy, and to assist consumers in distinguishing real health threats from spurious health claims...there is a strong scientific and medical foundation for THR, which shows great potential as a public health strategy to help millions of smokers." [95]

It is ironic that vocal and enthusiastic calls to implement THR have come from tobacco experts in New Zealand and Australia, where ST is effectively banned. Writing in the *New Zealand Medical Journal *in 2007, Laugesen urged government action: "Added to the mountain of evidence against cigarettes, sufficient evidence now exists for [the New Zealand] government to use [ST] to create safer tobacco choices for smokers, end cigarette sales altogether, and thus end the cigarette smoking deaths epidemic - in which 200,000 New Zealanders have died so far." [96]

Australian researchers Coral Gartner and Wayne Hall made an interesting comparison between ST use and alcohol consumption in a 2007 *Public Library of Science Medicine *article: "On current evidence the health risks of [ST] are comparable to those of regular alcohol use rather than cigarette smoking..."If the goal of tobacco control is to reduce tobacco-related disease, rather than tobacco use per se, then the promotion of [ST] use by inveterate smokers is a promising public health policy." [97]

In 2008 Gartner and Hall criticized the provision of misinformation by public health authorities in the U.S. and Australia in the *Medical Journal of Australia*: "Public health authorities in Australia and the United States have also claimed that SLT products: 'are just as bad for your health as cigarettes.' The epidemiological evidence shows that this is untrue. Dissemination by governments of misinformation on the relative harms of [ST] creates scepticism and mistrust of public health messages. It is paternalistic to misinform smokers about the risks of ST products for fear of increasing population nicotine use. We think it is also unethical to deny smokers access to a product that may reduce their health risk while cigarettes are readily available and very few quit attempts succeed." [98]

In 2007 a landmark report was published by the Royal College of Physicians, one of the oldest and most prestigious medical societies in the world [99]. Its findings were unequivocal: "Compiled by leading experts in the field, this report makes the case for harm reduction strategies to protect smokers. It demonstrates that smokers smoke predominantly for nicotine, that nicotine itself is not especially hazardous, and that if nicotine could be provided in a form that is acceptable and effective as a cigarette substitute, millions of lives could be saved."

In 2007, Foulds and Kozlowski provided a global perspective: "Around a billion people are addicted to nicotine in deadly cigarettes and many have no immediate plans to quit. Young people will also continue to try dangerous and addictive products. We believe it is preferable that, if people become addicted to cigarettes or decide to try tobacco, they can use a product that is markedly less harmful than cigarettes...we should not delay in allowing [ST] to compete with cigarettes for market share, and we should be prepared to accurately inform smokers about the relative risks of cigarettes, [ST], and approved smoking-cessation medications. In light of all the available evidence, the banning or exaggerated opposition to [ST] in cigarette-rife environments is not sound public-health policy." [100]

In 2008, Britton and Edwards lamented the lack of progress against smoking and urged governments to incorporate THR into tobacco regulatory frameworks: "In the 50 years since the health risks of smoking first became widely recognized, the political and public health responses to smoking at national and international levels have been grossly inadequate...A logical harm reduction approach for the millions of smokers who are unlikely to achieve complete abstinence...is to promote the substitution of tobacco smoking with an alternative, less hazardous means of obtaining nicotine...We believe that the absence of effective harm reduction strategies for smokers is perverse, unjust, and acts against the rights and best interests of smokers and the public health...The regulatory framework should therefore apply the levers of affordability, promotion, and availability in direct inverse relation to the hazard of the product, thus creating the most favourable market environment for the least hazardous products while also strongly discouraging use of smoked tobacco." [101]

In recent months government agencies in the U.S. and the United Kingdom (U.K.) have shown interest in long-term nicotine maintenance for smokers unwilling or unable to quit [102,103], a strategy described by Rodu and Cole in 1999 [104]. In October 2010 the U.S. FDA held a public workshop entitled "Risks and Benefits of Long-Term Use of Nicotine Replacement Therapy (NRT) Products" [102], which raises the prospect that the agency might eventually approve long-term use of pharmaceutical nicotine. It was noted at that workshop that epidemiologic studies of Swedish snus use provide most of the evidence for the minimal risks related to chronic nicotine use.

Similarly, in March 2011 the U.K. Department of Health released a white paper detailing "what the Government will do to support efforts to reduce tobacco use over the next five years..." [103]. This report noted that "the Medicines and Healthcare products regulatory agency granted an extended indication in 2010 for [pharmaceutical nicotine products] to be used for 'harm reduction', to assist smokers who are unwilling or unable to quit, as a safer alternative to smoking and to reduce the health hazards from secondhand smoke." In addition, the report stated that the government "will work in collaboration with the public health community to consider what more can be done to help tobacco users who cannot quit, or who are unwilling to, to substitute alternative safer sources of nicotine, such as [pharmaceutical nicotine], for tobacco. In support of this, [the National Institute for Health and Clinical Excellence] will produce public health guidance on the use of harm reduction approaches to smoking cessation (to be published in spring 2013). We will also encourage the manufacturers of safer sources of nicotine, such as [pharmaceutical nicotine], to develop new types of nicotine products that are more affordable and that have increased acceptability for use in the longer term."

Although the FDA workshop and the British white paper primarily focused on pharmaceutical nicotine, the implications are clear: tobacco harm reduction is on the horizon as a viable strategy in the U.S. and the U.K.

Sweanor et al. summarized the global public health implications of THR in a 2007 article in the *International Journal of Drug Policy*: "The relative safety of ST and other smokefree systems for delivering nicotine demolishes the claim that abstinence-only approaches to tobacco are rational public health campaigns...Applying harm reduction principles to public health policies on tobacco/nicotine is more than simply a rational and humane policy. It is more than a pragmatic response to a market that is, anyway, already in the process of undergoing significant changes. It has the potential to lead to one of the greatest public health breakthroughs in human history by fundamentally changing the forecast of a billion cigarette-caused deaths this century." [105]

## List of  Abbreviations

ACSH: American Council on Science and Health; aOR: adjusted odds ratio; EU: European Union; FDA: U.S. Food and Drug Administration; GI: gastrointestinal; HR: hazard ratio; LCMR: lung cancer mortality rate; MS: multiple sclerosis; NNAL: 4-(methylnitrosamino)-1-(3-pyridyl)-1-butanol; NNK: 4-(methylnitrosamino)-1-(3-pyridyl)-1-butanone; NNN: N'-nitrosonornicotine; OR: odds ratio; PET: positron emission tomography; RR: relative risk; ST: smokeless tobacco; THR: tobacco harm reduction; TSNA: tobacco-specific nitrosamine; US: United States; WHO: World Health Organization.

## Competing interests

The author's research is supported by unrestricted grants from Swedish Match AB, Reynolds American Inc. Services Company, Altria Client Services and British American Tobacco to the University of Louisville. He has no financial or other personal relationship with the sponsors or any other stakeholder in the issue of tobacco use and health.
